# Microbial Communities in Human Milk Relate to Measures of Maternal Weight

**DOI:** 10.3389/fmicb.2019.02886

**Published:** 2019-12-20

**Authors:** Sara N. Lundgren, Juliette C. Madan, Margaret R. Karagas, Hilary G. Morrison, Anne G. Hoen, Brock C. Christensen

**Affiliations:** ^1^Department of Epidemiology, Geisel School of Medicine at Dartmouth, Hanover, NH, United States; ^2^Institute for Molecular Medicine Finland, University of Helsinki, Helsinki, Finland; ^3^Division of Neonatology, Department of Pediatrics, Children’s Hospital at Dartmouth, Lebanon, NH, United States; ^4^Department of Community and Family Medicine, Geisel School of Medicine at Dartmouth, Hanover, NH, United States; ^5^Josephine Bay Paul Center, Marine Biological Laboratory, Woods Hole, MA, United States; ^6^Department of Biomedical Data Science, Geisel School of Medicine at Dartmouth, Hanover, NH, United States; ^7^Department of Molecular and Systems Biology, Geisel School of Medicine at Dartmouth, Hanover, NH, United States

**Keywords:** human milk, microbiome, BMI, gestational weight gain, 16S rRNA gene sequencing

## Abstract

The process of breastfeeding exposes infants to bioactive substances including a diversity of bacteria from breast milk as well as maternal skin. Knowledge of the character of and variation in these microbial communities, as well as the factors that influence them, is limited. We aimed to identify profiles of breastfeeding-associated microbial communities and their association with maternal and infant factors. Bilateral milk samples were collected from women in the New Hampshire Birth Cohort Study at approximately 6 weeks postpartum without sterilization of the skin in order to capture the infant-relevant exposure. We sequenced the V4–V5 hypervariable region of the bacterial 16S rRNA gene in 155 human milk samples. We used unsupervised clustering (partitioning around medoids) to identify microbial profiles in milk samples, and multinomial logistic regression to test their relation with maternal and infant variables. Associations between alpha diversity and maternal and infant factors were tested with linear models. Four breastfeeding microbiome types (BMTs) were identified, which differed in alpha diversity and in *Streptococcus*, *Staphylococcus*, *Acinetobacter*, and *Pseudomonas* abundances. Higher maternal pre-pregnancy BMI was associated with increased odds of belonging to BMT1 [OR (95% CI) = 1.13 (1.02, 1.24)] or BMT3 [OR (95% CI) = 1.12 (1.01, 1.25)] compared to BMT2. Independently, increased gestational weight gain was related to reduced odds of membership in BMT1 [OR (95% CI) = 0.66 (0.44, 1.00) per 10 pounds]. Alpha diversity was positively associated with gestational weight gain and negatively associated with postpartum sample collection week. There were no statistically significant associations of breastfeeding microbiota with delivery mode. Our results indicate that the breastfeeding microbiome partitions into four profiles and that its composition and diversity is associated with measures of maternal weight.

## Introduction

Through breastfeeding, infants are exposed to a diversity of bacteria from both maternal skin and milk (Pannaraj et al., 2017). Human milk is comprised of numerous bioactive substances including immune (Goldman et al., 1982; Hawkes et al., 1999; Admyre et al., 2007) and nutritive factors (López-López et al., 2002; Locascio et al., 2007) in addition to microbial communities. A distinct set of bacterial communities inhabits the breast skin, and both breast milk and skin-associated microbes are transferred to infants through the process of breastfeeding (Solis et al., 2010; Murphy et al., 2017; Pannaraj et al., 2017). The process of bacterial colonization of the gut in infancy is a key modulator of immune development (Grönlund et al., 2000; Mazmanian et al., 2005; Chichlowski et al., 2012) that may alter risk of early- and later-life diseases (Kalliomäki et al., 2008; Giongo et al., 2011; Arrieta et al., 2015). Breastfed infants have a reduced risk of diseases including otitis media, lower respiratory infections, gastroenteritis, atopy, cancers, and autoimmune disease, among others (Ip et al., 2007). The microbiota present in breast milk affect the symptomaticity of rotavirus infection in infants; infants with a symptomatic rotavirus infection consumed breast milk with lower levels of *Streptococcus* and *Staphylococcus* and higher levels of *Enterobacter* and *Klebsiella* compared to infants either negative for rotavirus or with an asymptomatic infection (Ramani et al., 2018). A more comprehensive understanding of factors influencing the microbiota present in breast milk and their contribution to the beneficial effects of breastfeeding has broad potential to influence public health.

Species of *Bifidobacterium*, *Streptococcus*, *Staphylococcus, Lactobacillus, Enterococcus*, and *Clostridia* have been observed in human milk, including butyrate-producing microbes (Martín et al., 2009; Cabrera-Rubio et al., 2012; Jost et al., 2013; Khodayar-Pardo et al., 2014). Butyrate is a short chain fatty acid of microbial metabolic origin that is known to be important for the integrity of the gut epithelial barrier and is protective against many intestinal and systemic diseases (Canani et al., 2011). Preliminary small studies using 16S rRNA gene sequencing and metagenomics approaches to assess breast milk microbial composition have reported that *Streptococcus*, *Staphylococcus*, *Pseudomonas*, *Bacteroides*, *Acinetobacter*, and others comprise the core breast milk microbiome, whereas *Bifidobacterium* and *Lactobacillus* abundances are comparatively low (Hunt et al., 2011; Jost et al., 2013; Ward et al., 2013; Jiménez et al., 2015; Sakwinska et al., 2016; Urbaniak et al., 2016; Murphy et al., 2017). Clusters of breast milk microbiota have been reported, often defined by the abundance of *Staphylococcus*, *Streptococcus*, and *Pseudomonas*, *Enterobacteriaceae*, and other rarer bacteria (Li et al., 2017; Biagi et al., 2018; Moossavi et al., 2019b).

The microbial composition of breast milk appears to be unique to each individual (Martín et al., 2007) and changes in early lactation, with bacterial counts rising with lactation stage (Khodayar-Pardo et al., 2014) and diversity increasing in the first 3 months postpartum (Simpson et al., 2018; Browne et al., 2019); however, bacterial diversity may be higher in colostrum compared to transition or mature milk (Cabrera-Rubio et al., 2012). Later in lactation, represented microbes appear stable within an individual in a single breast over a 4-week period of lactation (Hunt et al., 2011).

Previously, the breast milk microbiome composition has most commonly been associated with maternal and infant factors including maternal weight variables, delivery mode, and gestational age at delivery (Cabrera-Rubio et al., 2012; Collado et al., 2012; Khodayar-Pardo et al., 2014; Davé et al., 2016). Overweight women and those who gain excessive weight in pregnancy have been observed to harbor higher *Lactobacillus* and *Staphylococcus*, and lower *Bifidobacterium* counts in two prior studies (Cabrera-Rubio et al., 2012; Collado et al., 2012). In addition, maternal pre-pregnancy BMI also has been negatively associated with *Streptococcus* abundance, positively associated with overall microbial diversity (Davé et al., 2016) and diversity of Firmicutes, and negatively associated with the diversity of Proteobacteria (Moossavi et al., 2019b). In a small study of 18 Finnish women, milk from mothers who delivered by elective cesarean section had differential abundance of a number of microbes compared to those who delivered by non-elective cesarean section or vaginal delivery (Cabrera-Rubio et al., 2012). Further, in a study of 32 Spanish women, cesarean delivery was associated with increased bacterial counts in colostrum and transitional milk, and reduced likelihood of *Bifidobacterium* presence (Khodayar-Pardo et al., 2014). Grouping by both delivery mode and intrapartum antibiotic exposure shows that subjects cluster primarily by delivery mode, although both factors significantly associate with milk microbiome profile (Hermansson et al., 2019). Prophylactic intrapartum antibiotic exposure negatively associated with *Bifidobacterium* levels (Padilha et al., 2019b), yet is associated with increased microbial diversity and richness in breast milk (Hermansson et al., 2019). Additionally, women who delivered prematurely show lower abundance of *Bifidobacterium* (Khodayar-Pardo et al., 2014). However, other studies have not observed associations of delivery mode or gestational age with breast milk microbiota (Sakwinska et al., 2016; Urbaniak et al., 2016; Pannaraj et al., 2017; Ojo-Okunola et al., 2019). Relatively small sample size or unmeasured confounding factors in prior work may account for inconsistent results across studies and indicates the need for further investigation in larger studies.

Other important factors to the human milk microbiome have been identified, including the method of feeding, likely due to microbes from the infant oral cavity being transferred to the breast during feeding or microbes derived from the breast pump contaminating pumped milk. In a study of preterm infant–mother pairs, the microbial composition of breast milk changed after infants began direct feeding by latching (Biagi et al., 2018). Further, milk samples from mothers who sometimes fed infants indirectly via pumped milk show decreased alpha diversity, reduced prevalence of *Bifidobacterium*, and increased levels of *Enterobacteriaceae* and *Pseudomonadaceae* (Moossavi et al., 2019b). Additionally, ethnicity and geographical location (Kumar et al., 2016; Ojo-Okunola et al., 2019), diet (Padilha et al., 2019a), social network size of mother–infant pairs (Meehan et al., 2018), human milk oligosaccharides and other metabolites (Gómez-Gallego et al., 2018; Moossavi et al., 2019a), as well as maternal secretor status (Cabrera-Rubio et al., 2019) have been shown to affect microbial populations in breast milk.

To date, preliminary literature examining the microbiota associated with breastfeeding has been limited by study size, has largely focused on the abundance of specific microbes, and has given an incomplete picture of infants’ microbial exposure during breastfeeding due to sterile milk collection protocols. As part of a large pregnancy cohort study, we had the opportunity to characterize the “breastfeeding microbiome,” or the microbial communities that infants are exposed to during feeding. We used milk collected from a relatively racially homogeneous population of women in New Hampshire who provided milk samples around 6 weeks postpartum, without sterilization of the skin prior to collection in order to capture the full range of bacteria infants are exposed to during feeding. Using sequencing of the V4–V5 hypervariable region of the bacterial 16S rRNA gene, we sought to identify associations of hypothesized factors with the breastfeeding microbiota, including maternal pre-pregnancy BMI, gestational weight gain, delivery mode, and gestational age.

## Materials and Methods

### Study Population

Study subjects were from the New Hampshire Birth Cohort Study (NHBCS). Eligible participants for this birth cohort are pregnant women between the ages of 18 and 45 years who report using a private well for their home water source and are receiving prenatal care in clinics in New Hampshire, United States, as previously described (Gilbert-Diamond et al., 2011; Farzan et al., 2013). Subjects were recruited between approximately 24 and 28 weeks of gestation at routine prenatal visits. The Center for the Protection of Human Subjects at Dartmouth gave institutional review board approval, and all methods were performed according to guidelines. All subjects gave written informed consent for participation for themselves and their infants.

Telephone interviews conducted every 4 months ascertained breastfeeding and infant formula supplementation, as well as maternal medication use such as antibiotics. Mothers reporting any infant formula supplementation were considered to be combination feeding, while those reporting only breastfeeding were considered to be exclusively breastfeeding. Delivery mode, including elective vs. unplanned or emergent cesarean section, and peripartum antibiotic exposure were determined from maternal delivery records, while maternal ever smoking, race, education, relationship status, and parity were measured via a self-administered questionnaire upon study entry. Pre-pregnancy height and weight were determined either by questionnaire or from the medical record, if self-reported measures were unavailable, and were used to compute pre-pregnancy BMI. Gestational age was determined from prenatal medical records, estimated from date of last menstrual period and ultrasound. Weight gain during pregnancy and gestational diabetes was ascertained in a self-administered postpartum questionnaire.

### Sample Collection and DNA Extraction

Breast milk samples were collected at home by study participants from unsterilized bilateral breasts, with separate study-provided sterile collection bottles used for milk from each breast. To capture a representative portrait of infant exposure during breastfeeding we did not use a sterile collection protocol. Subjects provided a minimum of 18 mL and up to 80 mL of milk from each breast, with a median of 35 mL per breast. Samples were stored in the refrigerator at participants homes for up to approximately 1 day, brought in cold packs to the postpartum follow-up appointment (between 3.7 and 12 weeks after delivery), and immediately chilled. Samples were processed within 24 h of receipt. Before sample processing, the milk was mixed by inverting the collection tubes 10 times. 3 mL of milk from each breast were combined, centrifuged at 3396 rcf for 90 min at 4°C. Fat and supernatant were removed, and 150 μL of PBS was added to and mixed with the cell pellet. The cell suspension was transferred to a 1.5 mL tube and frozen at −20°C. The sample was thawed at room temperature, and microbial DNA was extracted using the Zymo DNA extraction kit (Zymo Research) and was quantified using the OD260/280 Nanodrop.

### Targeted 16S rRNA Sequencing

Using established methods (Huse et al., 2014; Newton et al., 2015), we sequenced the V4-V5 hypervariable region of the bacterial 16S rRNA gene at the Marine Biological Laboratory (MBL) in Woods Hole, MA, United States. Fusion primers were used to generate 16S rDNA V4–V5 amplicons from purified genomic DNA samples. Multiplexing of 96 samples per lane was possible due to the use of forward primers containing one of eight five-nucleotide barcodes between the Illumina-specific bridge and sequencing primer regions and the 16S-specific region and a single reverse primer containing one of 12 Illumina indices. Amplifications in triplicate with one negative control were used for internal quality control at the MBL in addition to positive and extraction controls prepared with samples at Dartmouth. The amplicon pool was quantified using qPCR (Kapa Biosystems). One Illumina MiSeq 500 cycle paired end run was used to sequence each pool of 96 libraries. Illumina MiSeq Reporter and a custom python script were used to demultiplex and divide datasets. Reads were not detected from extraction controls.

### Microbiome Profiling

Microbial population profiles were identified using full-length amplicon sequences for the V4–V5 hypervariable regions of the 16S rRNA gene. Forward and reverse reads were joined based on sequence overlap (Eren et al., 2013). Primer sequences were removed, and sequences with any ambiguous nucleotides were discarded. The vsearch program was implemented to remove both known chimeras in comparison to the RDP classifier training reference and *de novo* (Rognes et al., 2016).

### Data Processing and Analysis

Using QIIME version 1.9.1 (Caporaso et al., 2010b) we identified open reference operational taxonomic units (OTUs) at 97% similarity using the UCLUST algorithm (Edgar, 2010). Bacterial taxonomy was assigned using PyNAST alignment (Caporaso et al., 2010a) to the Greengenes reference database (Desantis et al., 2006; Mcdonald et al., 2012; Werner et al., 2012), and FastTree was used to construct the phylogenetic tree (Price et al., 2010). All statistical analyses were conducted in R version 3.3.0 (R Core Team, 2016).

Of 163 human milk samples collected at the 6-week time point and sequenced by September 24, 2016, 3 samples with less than 1000 sequence reads, 3 samples that were collected after 12 weeks postpartum, and 2 subjects with improbable weight gain values were excluded, resulting in a sample size of 155.

We calculated generalized UniFrac (GUniFrac) distances between paired samples using the OTU table, rarefied to the minimum sequencing depth (1863 reads), and midpoint rooted phylogenetic tree (Chen et al., 2012) to characterize the beta diversity of samples. Alpha diversity, a measure of the number and evenness of microbes in a sample, was characterized using Simpson’s diversity index, calculated using the *diversity* function in the R package *vegan* (Oksanen et al., 2015). Since Simpson’s diversity is bounded by 0 and 1, we logit transformed the values for analyses. We identified clusters of microbiota measured in human milk, “breastfeeding microbiome types (BMTs)” using an unsupervised clustering method, partitioning around medoids (PAM), on GUniFrac distances, as others have found this algorithm to perform well for microbiome data (Arumugam et al., 2011; Wu et al., 2011). We chose the optimal number of clusters (*k* = 4) by assessing within-cluster sum of squares for k of 2 through 10 using the elbow method, where the k at which the sum of squares begins to decrease less rapidly (the bend in the “elbow”) is considered optimal. Additionally, we examined average silhouette width (a measure of a sample’s similarity to its own versus another cluster), and observed separation of samples along the first and second principal coordinates by abundances of select bacterial taxa (*Streptococcus*, *Staphylococcus*, *Acinetobacter*, and *Pseudomonas*) and alpha diversity respectively. The mean silhouette width was greatest for *k* = 2, but the within cluster sum of squares continued to decrease until *k* = 5. This information, together with the observations related to cluster separation along the first axis by *Streptococcus* and *Staphylococcus* abundance, and along the second axis by alpha diversity, suggested *k* = 4 to be a biologically meaningful number of clusters. We compared alpha diversity between clusters using ANOVA and calculated 95% confidence intervals for pairwise comparisons using Tukey’s Honest Significant Difference. Differences in microbial community structure between clusters was tested using the permutational multivariate analysis of variance (PERMANOVA) method on GUniFrac distances with 10,000 permutations using the *adonis* function in the R package *vegan* (Oksanen et al., 2015). We tested for differences in the relative abundance of the top 25 most abundant (ranked by the median relative abundance) microbial taxa using the Kruskal–Wallis rank sum test, and Dunn’s test with FDR *p*-value correction to identify pairwise differences between BMTs. The Kruskal–Wallis rank sum test or Fisher’s exact test was used to test univariate associations between each variable and the BMT. Pairwise differences by BMT in maternal pre-pregnancy BMI and gestational weight gain were determined with Dunn’s test with FDR *p*-value correction. Multinomial logistic regression was used to test multivariate associations of covariates with cluster membership where the largest cluster (BMT2) was the reference. We calculated the predicted probabilities for each sample’s cluster membership, and plotted these values as a function of significant variables.

We used linear regression to test the associations between each variable and alpha diversity in a univariate and multivariate manner. Pairwise differences in alpha diversity by delivery mode were determined using Tukey’s Honest Significant Difference. To assess the relation between variables of interest and microbial community structure, we used the PERMANOVA method on GUniFrac distances with 10,000 permutations as specified previously.

For adjusted analyses, we limited our subjects to those without missing data for any variable with *p*-value < 0.10 in univariate analyses (*n* = 123). We performed sensitivity analyses limiting to women known to not have taken antibiotics in the postpartum period (up to 4-months postpartum, *n* = 92). We compared baseline characteristics between the overall study population and the subsets used for adjusted and sensitivity analyses (Supplementary Table S1).

## Results

### Subject Characteristics

Subject characteristics are presented in Table 1. Among study subjects, 94.8% were white, 81.3% were married, and average age was 32.4 years at study enrollment. Subjects were slightly overweight, with an average pre-pregnancy BMI of 26.0 and 41.3% gained more than the recommended amount of weight during pregnancy according to the Institute of Medicine guidelines (Institute of Medicine (US) and National Research Council (US) Committee to Reexamine Iom Pregnancy Weight Guidelines, 2009). Briefly, the recommended weight gain depends on pre-pregnancy BMI; for a singleton pregnancy, underweight, normal weight, overweight, and obese women should gain between 28–40, 25–35, 15–25, and 11–20 pounds, respectively. Parity ranged from 1 to 5. Gestational diabetes was rare, affecting only 5.2% of women, while peripartum antibiotic exposure was common with 45.8% of women given antibiotics before, during, or immediately after labor. Antibiotic exposure occurred in around 15% of subjects prenatally, and 11% within the first 4 months postpartum. Most deliveries were full term (average 39.2 weeks), but ranged from 30.0 to 42.4 weeks. The majority of women (70.3%) were exclusively breastfeeding at the time of sample collection around 6-weeks postpartum.

**TABLE 1:** Subject characteristics (*N* = 155).

**Variable**	**N (%) or Mean [Range]**
Age	32.4 [20–45]
Race^a^	
White	147 (94.8)
Other	3 (1.9)
Relationship status^b^	
Married	126 (81.3)
Separated or divorced	4 (2.6)
Single and never married	12 (7.7)
Smoking history^c^	
No	126 (81.3)
Yes	15 (9.7)
Prepregnancy BMI^d^	26.0 [17.4–47.8]
Weight gain during pregnancy (lbs)^d^	32.7 [4–63]
Weight gain category^e^	
Less	23 (14.8)
Recommended	36 (23.2)
More	64 (41.3)
Parity^f^	1.8 [1–5]
Delivery mode^b^	
Vaginal	101 (65.2)
Elective Cesarean section	22 (14.2)
Non-elective Cesarean section	19 (12.3)
Gestational diabetes^g^	
No	125 (80.6)
Yes	8 (5.2)
Maternal prenatal antibiotics^h^	
No	96 (61.9)
Yes	23 (14.8)
Maternal antibiotics by 4-months postpartum^i^	
No	113 (72.9)
Yes	17 (11.0)
Peripartum antibiotics^j^	
No	69 (44.5)
Yes	71 (45.8)
Infant feeding behavior^k^	
Exclusively breastfed	109 (70.3)
Combination fed	22 (14.2)
Gestational age at delivery	39.2 [30.0–42.4]
Infant sex^l^	
Female	73 (47.1)
Male	74 (47.7)
Postpartum collection week	6.6 [3.7–12.0]

*Footnotes indicate the number of subjects with missing values: ^*a*^5, ^*b*^13, ^*c*^14, ^*d*^17, ^*e*^32, ^*f*^3, ^*g*^22, ^*h*^36, ^*i*^25, ^*j*^15, ^*k*^24, ^*l*^8.*

### Most Abundant Breast Milk Taxa and Breast Milk Microbiome Types

High-throughput sequencing of the bacterial 16S rRNA gene V4–V5 hypervariable region yielded an average of ∼75,000 sequence reads per sample and a median of 46,366 reads per sample, with a range from 1,863 to 1,234,419 (Supplementary Figure S1). To ensure the largest possible sample size, we rarefied to the minimum sequencing depth, with reads mapping to 4,108 OTUs. The most highly abundant taxon was *Acinetobacter*, accounting for 14.3% of sequence reads per sample on average, followed by *Streptococcus* (13.7%), *Pseudomonas* (11.3%), *Staphylococcus* (11.0%), and *Enterobacteriaceae* (8.23%). *Bacteroides* and *Bifidobacterium*, well-studied microbes in the context of the infant gut, were present at 1.07 and 0.651% respectively.

**FIGURE 1:**
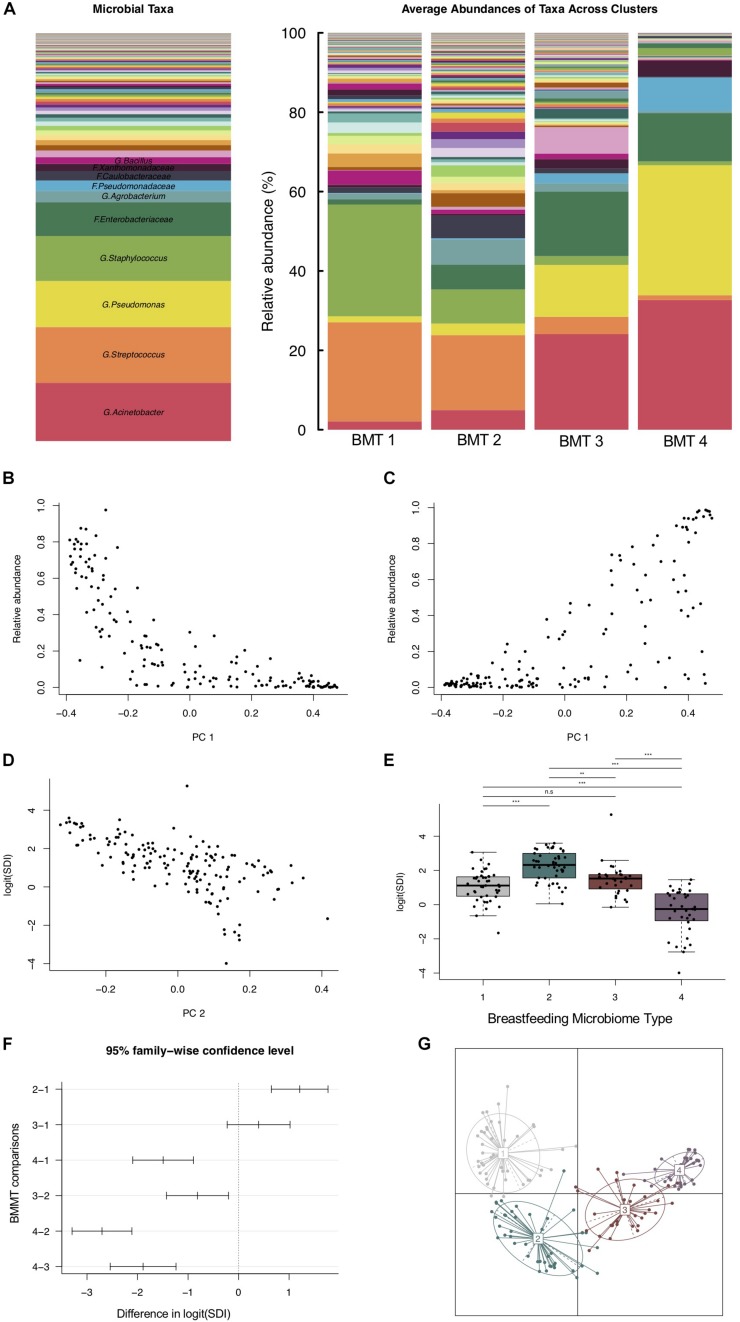
Breastfeeding microbiome types (BMTs) differ in diversity and taxonomic abundances. **(A)** Barplot showing the relative abundance of microbial taxa overall and on average within each of the four breastfeeding microbiome types [BMT1 (*n* = 43), BMT2 (*n* = 46), BMT3 (*n* = 31), BMT4 (*n* = 35)]. **(B)** The y-axis represents the combined relative abundance of *Streptococcus* + *Staphylococcus* in breast milk samples as a function of PC 1. **(C)** The y-axis represents the combined relative abundance of *Pseudomonas* + *Acinetobacter* in breast milk samples as a function of PC 1. **(D)** Microbial alpha diversity (Simpson’s diversity index) as a function of PC 2. **(E)** Boxplot showing microbial alpha diversity for each of the four BMTs, with significance of pairwise differences determined by ANOVA and Tukey’s HSD. Significance levels are n.s., not significant, ^∗^*p*-value < 0.05, ^∗∗^*p*-value < 0.01, ^∗∗∗^*p*-value < 0.001. **(F)** 95% confidence interval for pairwise differences in alpha diversity between BMTs calculated using ANOVA and Tukey’s HSD. **(G)** Principal coordinate analysis plot colored by BMT, indicated by the number label at the cluster centroid. Each point represents one subject, and lines indicate the distance from the cluster centroid.

**FIGURE 2:**
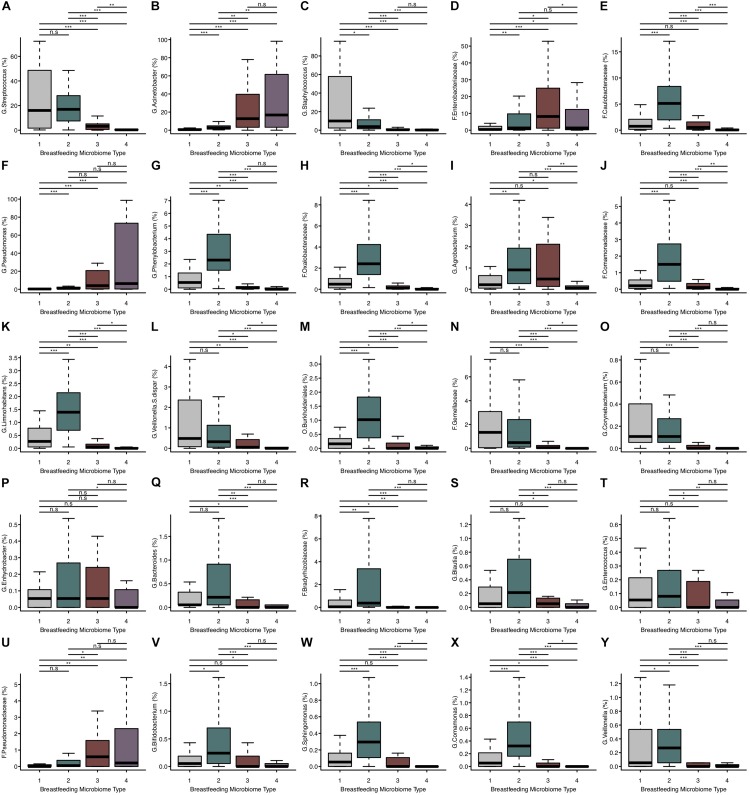
The relative abundance of microbial taxa differs between BMTs. **(A–Y)** Boxplots showing the relative abundance of the top 25 most abundant microbial taxa in breast milk for each BMT. Significance was determined by the Kruskal-Wallis rank-sum test followed by Dunn’s test with FDR correction. Significance levels are n.s., not significant, ^∗^*p*-value < 0.05, ^∗∗^*p*-value < 0.01, ^∗∗∗^*p*-value < 0.001.

Using partitioning around medoids, we identified four clusters of human milk microbiota, which we refer to as BMTs (Figure 1A). In principal coordinates analysis, PC 1 was positively related to abundance of *Acinetobacter* and *Pseudomonas* (Figure 1C), and negatively related to *Streptococcus* and *Staphylococcus* abundance (Figure 1B), while PC 2 was negatively related to Simpson’s diversity (Figure 1D). BMT1 was characterized by a high abundance of *Streptococcus* and *Staphylococcus* combined (>50% of a sample on average). *Staphylococcus* was highest and *Enterobacteriaceae* and *Pseudomonas* were lowest in BMT1 compared to all other clusters (Figures 2C,D). In BMT2 *Streptococcus* was the most abundant taxon, but samples were overall more diverse than those in BMT1, with other bacterial taxa comprising a large portion of microbiota, such as *Caulobacteraceae* with a median relative abundance of over 5%, and *Phenylobacterium* and *Oxalobacteraceae* with median relative abundances over 2% (Table 2); additionally, *Bifidobacterium* was most abundant in BMT2 compared to all other clusters (Figure 2V). Both BMT1 and BMT2 had higher *Bacteroides* levels than BMT3 and BMT4 (Figure 2Q). *Acinetobacter* was predominant in BMTs 3 and 4, with BMT3 also showing high median abundances of *Enterobacteriaceae* and *Pseudomonas* of around 8 and 4%, respectively. BMT4 was the least diverse cluster (Figure 1F) and was comprised predominantly of *Acinetobacter*, followed by *Pseudomonas* (Table 2). Simpson’s diversity index was highest in BMT2, and lowest in BMT4 (Figure 1E); all clusters had significantly different alpha diversity except for BMT1 and BMT3 (Figure 1F). Overall microbial community structure was significantly different according to BMT cluster membership (Figure 1G, GUniFrac PERMANOVA *p*-value = 1.0 × 10^–4^).

**TABLE 2:** Median relative abundance of microbial taxa differs between BMTs.

	**Median relative abundance,% (IQR)**					***p*-value^b^**
		
**Microbial taxon^a^**	**Overall (*N* = 155)**	**BMT1 (*N* = 43)**	**BMT2 (*N* = 46)**	**BMT3 (*N* = 31)**	**BMT4 (*N* = 35)**	
*G.Streptococcus*	4.4(0.62−19.59)	16.05(1.69−48.63)	16.94(8.21−27.87)	3.17(0.59−5.15)	0.32(0.05−0.72)	5.10E-15
*G.Acinetobacter*	2.52(0.75−12.88)	0.64(0.24−1.53)	2.58(1.19−4.75)	12.72(3.22−39.64)	16.8(3.25−61.62)	2.70E-10
*G.Staphylococcus*	1.99(0.32−8.32)	9.93(2.39−57.84)	3.84(1.56−10.99)	0.48(0.19−1.45)	0.21(0.05−0.89)	7.20E-13
*F.Enterobacteriaceae*	1.4(0.21−8.86)	0.43(0.16−2.33)	1.34(0.32−9.68)	8.16(1.21−24.99)	1.34(0.3−12.35)	1.60E-04
*F.Caulobacteraceae*	0.81(0.13−3.09)	0.75(0.16−2.09)	5.13(2.03−8.35)	0.54(0.16−1.58)	0.05(0−0.19)	1.60E-17
*G.Pseudomonas*	0.81(0.32−4.83)	0.32(0.13−0.64)	0.97(0.59−2.35)	4.03(0.38−20.75)	6.33(0.32−73.3)	1.50E-06
*G.Phenylobacterium*	0.38(0.05−1.72)	0.54(0.11−1.29)	2.31(1.52−4.29)	0.11(0.05−0.21)	0(0−0.11)	1.20E-19
*F.Oxalobacteraceae*	0.38(0.05−1.45)	0.48(0.13−1.02)	2.42(1.4−4.17)	0.11(0.05−0.32)	0(0−0.08)	3.90E-21
*G.Agrobacterium*	0.32(0.05−1.13)	0.21(0.05−0.64)	0.91(0.28−1.92)	0.48(0.13−2.12)	0.05(0−0.16)	2.80E-06
*F.Comamonadaceae*	0.27(0−0.83)	0.21(0.05−0.56)	1.5(0.5−2.72)	0.11(0.03−0.35)	0(0−0.05)	3.70E-18
*G.Limnohabitans*	0.16(0−0.86)	0.27(0.05−0.78)	1.4(0.71−2.09)	0.05(0−0.16)	0(0−0.03)	9.00E-22
*G.Veillonella.S.dispar*	0.11(0−0.67)	0.48(0.08−2.36)	0.32(0.05−1.11)	0.05(0−0.43)	0(0−0.05)	1.10E-08
*O.Burkholderiales*	0.11(0−0.56)	0.16(0−0.35)	1.02(0.39−1.83)	0(0−0.19)	0(0−0.05)	3.10E-17
*F.Gemellaceae*	0.11(0−1.07)	1.34(0.05−3.09)	0.48(0.11−2.31)	0.05(0−0.24)	0(0−0)	5.20E-11
*G.Corynebacterium*	0.05(0−0.16)	0.11(0.05−0.4)	0.11(0.05−0.25)	0(0−0.03)	0(0−0)	2.40E-09
*G.Enhydrobacter*	0.05(0−0.16)	0.05(0−0.11)	0.05(0.01−0.27)	0.05(0−0.24)	0(0−0.11)	0.051
*G.Bacteroides*	0.05(0−0.38)	0.05(0.05−0.32)	0.21(0.05−0.89)	0(0−0.16)	0(0−0.05)	1.80E-05
*F.Bradyrhizobiaceae*	0.05(0−0.3)	0.05(0−0.64)	0.38(0.11−3.14)	0(0−0.05)	0(0−0)	1.60E-09
*G.Blautia*	0.05(0−0.27)	0.05(0−0.3)	0.21(0−0.68)	0.05(0−0.13)	0(0−0.05)	5.20E-04
*G.Enterococcus*	0.05(0−0.21)	0.05(0−0.21)	0.08(0−0.25)	0(0−0.19)	0(0−0.05)	8.50E-03
*F.Pseudomonadaceae*	0.05(0−0.48)	0(0−0.11)	0.05(0−0.36)	0.59(0−1.58)	0.21(0−2.31)	1.40E-03
*G.Bifidobacterium*	0.05(0−0.3)	0.05(0−0.19)	0.24(0.05−0.67)	0(0−0.19)	0(0−0.05)	4.30E-05
*G.Sphingomonas*	0.05(0−0.27)	0.05(0−0.16)	0.3(0.11−0.51)	0(0−0.11)	0(0−0)	1.20E-12
*G.Comamonas*	0.05(0−0.27)	0.05(0−0.21)	0.32(0.17−0.64)	0(0−0.05)	0(0−0)	8.70E-16
*G.Veillonella*	0.05(0−0.27)	0.05(0−0.54)	0.27(0.05−0.52)	0(0−0.05)	0(0−0.03)	8.80E-08

*^*a*^*F*., *G.*, and *S.* indicate the level of taxonomy to be family, genus, or species. ^*b*^*p*-values were calculated using the Kruskal–Wallis rank-sum test.*

There were both similarities and differences in the relative abundances of microbial taxa between different BMTs (Figures 2A–Y). For example, BMT1 and BMT2 had similar relative abundances of *Streptococcus* (Figure 2A) and *Bacteroides* (Figure 2Q), yet differed in abundances of *Staphylococcus* (Figure 2C), *Enterobacteriaceae* (Figure 2D), *Bifidobacterium* (Figure 2V) and many other taxa. Similarly, BMT3 and BMT4 had similar levels of *Acinetobacter* (Figure 2B) and *Pseudomonas* (Figure 2F), but differed by *Streptococcus* (Figure 2A), *Caulobacteraceae* (Figure 2E), and other taxa. Abundances of most taxa were different when comparing BMT1 to BMT3 and BMT4, except for a few taxa including *Caulobacteraceae* (BMT3, Figure 2E), and *Enhydrobacter* (BMT4, Figure 2P). Likewise, BMT2 was mostly different from BMT3 and BMT4, except for in the abundance of a few taxa. The relative abundances of some taxa, including *Oxalobacteraceae*, *Limnohabitans*, and *Burkholderiales*, were significantly different between all BMTs (Figures 2H,K,M).

**FIGURE 3:**
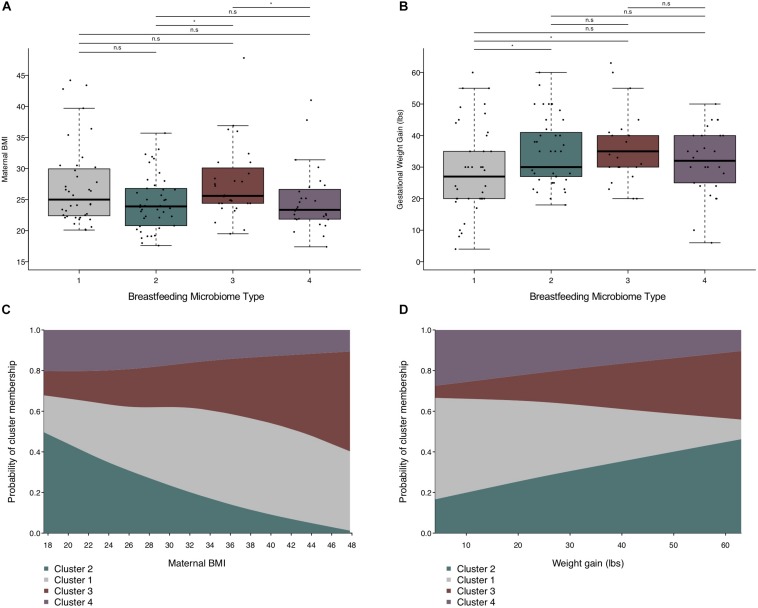
Maternal weight measures are related to BMT. **(A)** Boxplot showing the distribution of maternal pre-pregnancy BMI by BMT. **(B)** Boxplot of the distribution of gestational weight gain by BMT. Significance was determined by the Kruskal–Wallis rank-sum test followed by Dunn’s test with FDR correction. Significance levels are n.s., not significant, ^∗^*p*-value < 0.05. **(C)** Predicted probability plot of BMT membership by pre-pregnancy BMI from multinomial logistic regression models (*n* = 123), where the proportion of each block along the y-axis represents the probability of cluster membership for a given pre-pregnancy BMI. The model was adjusted for sample collection week, weight gain during pregnancy (per 10 lbs.), and parity. Cluster 2 is the reference group. **(D)** Predicted probability plot of BMT membership by weight gain during pregnancy from multinomial logistic regression models (*n* = 123), where the proportion of each block along the y-axis represents the probability of cluster membership for a given gestational weight gain. The model was adjusted for sample collection week, pre-pregnancy BMI, and parity. Cluster 2 is the reference group.

In univariate analyses, maternal pre-pregnancy BMI was associated with cluster membership (Kruskal–Wallis rank sum test *p*-value = 0.042, *n* = 138), primarily between BMT2 vs. BMT3 (Dunn’s test *p*-value < 0.05) and BMT2 vs. BMT4 (Dunn’s test *p*-value < 0.05). Weight gain during pregnancy was marginally associated with cluster membership overall (Kruskal–Wallis rank sum test *p*-value = 0.050, *n* = 138), but significant differences were detected for BMT1 vs. BMT2 (Dunn’s test *p*-value < 0.05) and BMT1 vs. BMT3 (Dunn’s test *p*-value < 0.05, Figure 3 and Table 3). In addition to maternal BMI and weight gain, univariate multinomial logistic regression provided evidence that parity and postpartum week at sample collection were related to cluster membership (Supplementary Table S2). To test for associations between covariates and BMTs we fit multinomial logistic regression models adjusted for maternal BMI, weight gain during pregnancy, parity, and postpartum week at sample collection with BMT2 as the reference cluster (*n* = 123, Supplementary Table S2). For each unit increase in maternal BMI we observed an increased odds of membership in BMT1 compared to BMT2 [OR (95% CI) = 1.13 (1.02, 1.24)] and BMT3 compared to BMT2 [OR (95% CI) = 1.12 (1.01, 1.25), Figure 3C]. Additionally, increasing maternal weight gain (per 10 pounds) was associated with decreased probability of belonging to BMT1 versus BMT2 [OR (95% CI) = 0.66 (0.44, 1.00)] (Figure 3D), and there was an increased probability membership in BMT3 versus BMT2 with increasing parity [OR (95% CI) = 1.57 (0.81, 3.04)] and of membership in BMT4 versus BMT2 with increasing sample collection week [OR (95% CI) = 1.72 (1.00, 2.95)], but these associations did not reach statistical significance in the adjusted model.

**TABLE 3:** Univariate associations with BMT.

**Variable**	***p*-value^a^**
Age	0.41
Prepregnancy BMI	0.042
Weight gain during pregnancy (lbs)	0.050
Parity	0.15
Delivery mode	0.45
Maternal prenatal antibiotics	0.87
Maternal peripartum antibiotics	0.28
Maternal antibiotics by 4-months postpartum	0.97
Infant feeding behavior	0.60
Gestational age at delivery	0.69
Infant sex	0.99
Postpartum collection week	0.13

*^*a*^*p*-values calculated using Fisher’s exact test or Kruskal–Wallis rank-sum test.*

### Characteristics Related to Microbial Alpha Diversity

**FIGURE 4:**
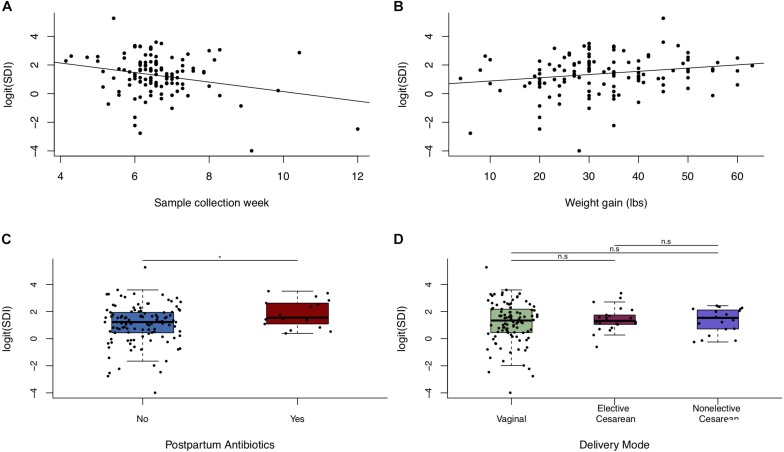
Alpha diversity of breastfeeding microbiota in relation to maternal weight and delivery mode. Scatter plots (*n* = 123) of logit(SDI) vs. **(A)** postpartum week at sample collection week and **(B)** weight gain during pregnancy. **(C)** Boxplot of logit(SDI) vs. postpartum antibiotic exposure. **(D)** Boxplot of logit(SDI) vs. delivery mode (*n* = 123). Significance was determined by ANOVA followed by Tukey’s HSD. Significance levels are n.s., not significant, ^∗^*p*-value < 0.05.

Univariate models showed a negative association between postpartum week at sample collection and breastfeeding microbiota alpha diversity, and a positive association of weight gain during pregnancy with alpha diversity (Figures 4A,B and Supplementary Table S3). Additionally, maternal antibiotic exposure by 4-months postpartum was positively associated with microbial alpha diversity (Figure 4C and Supplementary Table S3), despite potential misclassification of exposure since we do not know if milk collection happened before or after antibiotic usage. Prenatal antibiotic exposure was not associated with breastfeeding microbiota diversity (Supplementary Table S3). In adjusted models, postpartum week at sample collection was associated with decreased alpha diversity (Simpson’s diversity, β = −0.33, *p*-value = 0.0039), and weight gain during pregnancy (per each 10 pounds gained) was associated with increased alpha diversity (Simpson’s diversity, β = 0.23, *p*-value = 0.022; Table 4). Restricting the analysis to women known to have not taken antibiotics before 4-months postpartum did not qualitatively change these results (Table 4). We found no clear association between alpha diversity of breastfeeding microbiota and delivery mode (Figure 4D and Supplementary Table S3), or between alpha diversity and women who experienced labor prior to delivery versus those that did not (data not shown).

**TABLE 4:** Alpha diversity of breastfeeding microbiota linear model results.

**Variable**	**Coefficient^a^**	***p*-value^a^**
Weight gain during pregnancy (per 10 lbs)	0.23	0.022
Postpartum collection week	–0.33	0.0039

**Variable**	**Coefficient^b^**	***p*-value^b^**

Weight gain during pregnancy (per 10 lbs)	0.31	0.0076
Postpartum collection week	–0.31	0.016

*^*a*^Analyses adjusted for weight gain during pregnancy and postpartum collection week (*n* = 123). ^*b*^Analyses restricted to women know to have not received antibiotics before 4 months postpartum, and adjusted for weight gain during pregnancy and postpartum collection week (*n* = 92).*

### Characteristics Related to Microbial Community Structure

Postpartum week at sample collection, maternal weight gain, and parity were marginally associated with microbial community structure of breastfeeding microbiota in PERMANOVA analysis on generalized UniFrac distances in both univariate models and those adjusted for sample collection age, maternal weight gain, and parity (Supplementary Table S4). Differences were not observed with the community structure of breastfeeding microbiota by delivery mode or by antibiotic exposure at any time. The association with maternal weight gain was significant in sensitivity analysis restricting to women known not to have received antibiotics by 4 months postpartum (GUniFrac PERMANOVA, *p*-value = 0.036).

## Discussion

We collected milk from women enrolled in a large molecular epidemiology birth cohort at approximately 6 weeks postpartum and measured the microbial composition using targeted sequencing of the 16S rRNA gene to evaluate physiological and environmental factors associated with the microbiota infants consume during feeding. Overall, the bacterial taxa we identified in our study are consistent with what has been found in the limited number of previous studies on human milk using sequencing of the bacterial 16S rRNA gene, with the most abundant bacteria on average belonging to the genera *Acinetobacter, Streptococcus*, *Pseudomonas*, and *Staphylococcus*. Unsupervised clustering revealed four main patterns of microbiota that have not previously been reported, denoted here as BMTs. While the average abundance of these four microbes are similar across all samples, two clusters each were characterized by higher abundances of *Streptococcus* and *Staphylococcus*, or *Pseudomonas* and *Acinetobacter* with differences in alpha diversity and the abundances of numerous other taxa further differentiating these clusters. The clusters we identified share both similarities and differences with the breast milk microbiome clusters identified in Chinese (Li et al., 2017), Italian (Biagi et al., 2018), and Canadian (Moossavi et al., 2019b) mothers, suggesting that some patterns of microbial populations in human milk may be similar amongst geographically and racially distinct populations.

Bacteria belonging to the genera *Acinetobacter* and *Pseudomonas* are often thought of as pathogens contributing to nosocomial infections (Fazeli et al., 2012; Custovic et al., 2014). However, given that over 40% of subjects belong to clusters characterized by high levels of these two microbes, we suggest their presence in breast milk is unlikely to be pathogenic. In fact, skin colonization of *Acinetobacter* has been associated with protection from allergies (Hanski et al., 2012) through dendritic cell programing (Debarry et al., 2010), a benefit that could possibly be transferred to the infant. In our cohort, 100% of infants with detectable *Acinetobacter* in their stool have mothers with detectable *Acinetobacter* in their milk, with an average relative abundance of 0.01% on average in the infant gut at 6-weeks of age. However, with targeted 16S rRNA sequencing data, we cannot confirm that the sequencing reads originate from the same strain in paired maternal milk and infant stool. Breast milk microbiota, including *Acinetobacter*, may contribute to the infant gut microbiome through multiple mechanisms in addition to direct colonization of the infant gut, for example through interactions with other microbes in the infant gut.

In the first BMT, Firmicutes were particularly prominent, a phylum associated with overweight and obesity (Koliada et al., 2017). This is interesting given that membership in the first cluster was associated with increased maternal pre-pregnancy BMI in our study. Studies suggest that maternal weight influences child weight status, potentially through alterations to infant gut microbiota (Ajslev et al., 2011; Mueller et al., 2016) in addition to genetics (Pietiläinen et al., 1999). Future studies may help elucidate whether infants of mothers belonging to this first cluster are at an increased risk for overweight.

We found a positive relationship between weight gain during pregnancy and bacterial alpha diversity of breastfeeding microbiota, as well as associations with cluster membership and microbial community structure. To our knowledge, this question has not previously been investigated in a cohort of this size. The maternal gut microbiome is thought to be a source of human milk microbiota (Jost et al., 2014), though only a small number of bacteria are shared between the maternal gut and breast milk (Avershina et al., 2018), and maternal intestinal microbiota have been found to correlate with weight gain during pregnancy (Santacruz et al., 2010). There is evidence that the maternal intestinal microbiome transforms in late pregnancy to promote insulin insensitivity, inflammation, and weight gain, and that this shift varies widely between women (Koren et al., 2012). It is possible that the higher alpha diversity of breastfeeding microbiota in women who gain more weight during pregnancy could result from this shift in maternal intestinal microbiota, perhaps due to changes in intestinal permeability. However, further studies are needed to test this hypothesis. Although maternal pre-pregnancy BMI was related to cluster membership in our study, we did not find an association with other measures of the microbiome. There was not strong evidence of a relation between parity and breastfeeding microbiota, or of infant gestational age, prenatal antibiotics, and peripartum antibiotics, although we found evidence that postpartum antibiotics relate with increased microbial diversity in breast milk despite potential misclassification of subjects. This suggests that the effect of antibiotics may be transient as is supported by existing studies (Padilha et al., 2019b), and that the effect of peripartum antibiotics may only be observable in the postpartum period before when the samples in this study were collected. Additional investigation will be necessary to understand these questions.

Delivery mode was not related to the breastfeeding microbiota in our study. Differences by delivery mode were observed in a study of milk samples from 80 women in four countries on three continents, but the effect was primarily among the subgroup of 20 Spanish women (Kumar et al., 2016). Consistent with our findings, a recent study of human milk from 60 Chinese women across three time points and two collection techniques (Sakwinska et al., 2016) and one studying 109 American mothers (Pannaraj et al., 2017) did not observe an association of cesarean section delivery with microbial populations in human milk. Other studies suggesting an influence of delivery mode on human milk microbiota (Cabrera-Rubio et al., 2012; Khodayar-Pardo et al., 2014; Cabrera-Rubio et al., 2016) were very small, with 32 subjects or fewer. Given the inter-individual variability of human milk microbiota, the addition of only a few subjects could potentially shift the results when the sample size is small, or the limited ability to adjust for confounding factors may account for varying findings between previous studies. Alternatively, differences in human milk microbiota by delivery mode may exist only early in the lactation period, dissipating by the time of sample collection in our study.

Many samples in our study contained *Acinetobacter*. This may be partially attributable to the collection method that did not involve sterilization of the breast skin prior to collection using a breast pump (Sakwinska et al., 2016), which we chose in order to have a clearer understanding of infants’ actual exposures. However, many subjects had very low abundances of this microbe despite the same collection method, so factors other than this likely contribute to its presence. Breastfeeding is not an aseptic process, and the microbial flora inhabiting the mother’s skin that infants are exposed to during feeding also play an important role in microbial colonization of the infant gut (Pannaraj et al., 2017). Non-aseptic human milk collection is thus preferable for assessing the relation between human milk and the infant gut microbiome, particularly since there is clear variation of these microbes between women. It is also possible that differences in both the detection of certain bacterial taxa as well as in reported associations between breast milk microbiota and factors of interest occur due to differences in the microbial sequencing method used. The reported 16S rRNA gene regions sequenced in reported studies varies widely, including V4–V5 used here, V1–V3 (Cabrera-Rubio et al., 2019), V1–V2 (Cabrera-Rubio et al., 2012), and commonly V4 (Kumar et al., 2016; Moossavi et al., 2019b; Ojo-Okunola et al., 2019) and others. In breast milk specifically, sequencing of the V1-V2 region does not detect *Lactobacillus* and *Acinetobacter*, V4 does not detect *Listeria*, V5-V6 does not detect *Staphylococcus* and *Veillonella*, and V9 does not detect *Streptococcus* (Meyer et al., 2019).

A strength of our study is its large sample size, which enabled us to adjust for potential confounding factors. Further, our well-defined, racially homogeneous study population supports the internal validity of our study. For instance, all of the subjects in our study resided in the same geographical region, and nearly all were white. Nonetheless, the generalizability of our findings to other populations requires independent validation. A high proportion of subjects in our study received peripartum antibiotics either as prophylaxis for cesarean delivery or for Group B *Streptococcus* colonization and peripartum prophylaxis, or for presumed intrapartum infection, which may differ in other populations. Our measure of maternal pre-pregnancy BMI is based on self-reported height and weight; at an average of 26.5 was lower than the U.S. average for women in 2015–2016 of 29.6 (Fryar et al., 2018). Given that 69% of subjects in our study completed college or postgraduate school (Emeny et al., 2019) and the association of high educational attainment with lower BMI (Hermann et al., 2011), it is not unexpected that BMI in our source population is lower than the U.S. national average. However, some subjects may have mis-reported their weight or height. Further, a pre-pregnancy measurement may not be the most relevant time of measurement for breastfeeding, which is occurs after delivery. Additionally, we were unable to adjust for maternal antibiotic usage that may have occurred in the approximately 6 week interval between delivery and sample collection due to lack of detail regarding the timing of exposure, but sensitivity analyses excluding individuals who may have received antibiotics in the first 4 months following delivery yielded consistent estimates.

## Conclusion

In a prospective pregnancy cohort study of the general population, we found that breastfeeding microbiota follow four general patterns characterized by high or low alpha diversity as well as differential abundances of *Streptococcus*, *Staphylococcus*, *Acinetobacter*, and *Pseudomonas*. We found that these patterns may be shaped by maternal weight, including both pre-pregnancy BMI and weight gain during pregnancy, independently. Future studies are necessary to determine the extent to which these microbial populations can be altered and the implications of milk microbiota to infants’ health, including the development of the infant intestinal microbiome.

## Data Availability Statement

The 16S rRNA gene sequences analyzed in this study are available through the National Center for Biotechnology Information (NCBI) Sequence Read Archive: http://www.ncbi.nlm.nih.gov/sra (Accession number PRJNA296814).

## Ethics Statement

The studies involving human participants were reviewed and approved by the Center for the Protection of Human Subjects at Dartmouth. The participants provided their written informed consent to participate in this study.

## Author Contributions

MK and JM designed the research. SL performed statistical analyses and wrote the manuscript. SL, AH, BC, MK, and JM interpreted the results. HM provided technical and methodological support from the Marine Biological Laboratory. BC has primary responsibility for the final content. All authors reviewed the manuscript, offered critical feedback, and approved the final version.

## Conflict of Interest

The authors declare that the research was conducted in the absence of any commercial or financial relationships that could be construed as a potential conflict of interest.
