# Supplementary material for: Microbial Communities in Human Milk Relate to Measures of Maternal Weight
Source: Front Microbiol. 2019 Dec 20;10:2886. doi: 10.3389/fmicb.2019.02886 (PMC6933483; doi:10.3389/fmicb.2019.02886)
Supplement: Supplementary file 1 [file Image_1.PDF]

Supplementary Material

1 Supplementary Figures and Tables

1.1 Supplementary Figures

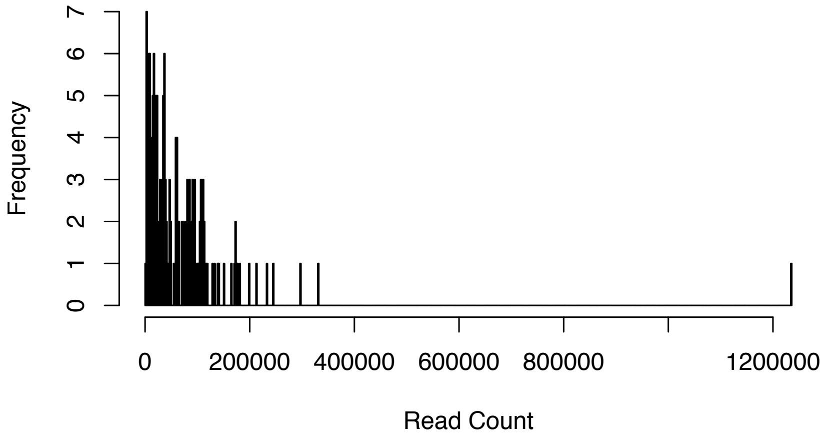

Supplementary Figure 1. Histogram of read counts per sample for microbial sequencing data.

1.2 Supplementary Tables.

Table S1. Comparison of subject characteristics between all study subjects and subsets used for multivariate and sensitivity analyses.

	N = 155		*N = 123*		*N = 92*	
**Variable[a]**	**N (%) or Mean [Range]**	**NA**	**N (%) or Mean [Range]**	**NA**	**N (%) or Mean [Range]**	**NA**
Age	32.4 [20 - 45]		32.7 [23 - 44]		32.9 [23 - 44]	
Race		5		1		
White	3 (1.9)		2 (1.6)		2 (2.2)	
Other	147 (94.8)		120 (97.6)		90 (97.8)	
Relationship status		13		6		2
Married	126 (81.3)		106 (86.2)		84 (91.3)	
Separated or divorced	4 (2.6)		4 (3.3)		2 (2.2)	
Single and never married	12 (7.7)		7 (5.7)		4 (4.3)	
Smoking history		14		7		2
No	126 (81.3)		105 (85.4)		82 (89.1)	
Yes	15 (9.7)		11 (8.9)		8 (8.7)	
Prepregnancy BMI	26 [17.4 - 47.8]	17	26.1 [17.6 - 47.8]		26.1 [18 - 47.8]	
Weight gain during pregnancy (lbs)	32.7 [4 - 63]	17	32.7 [4 - 63]		32.5 [4 - 63]	
Weight gain category		32				
Less	23 (14.8)		23 (18.7)		20 (21.7)	
Recommended	36 (23.2)		36 (29.3)		26 (28.3)	
More	64 (41.3)		64 (52)		46 (50)	
Parity	1.8 [1 - 5]	3	1.7 [1 - 5]		1.7 [1 - 5]	
Delivery mode		13		6		4
Vaginal	101 (65.2)		80 (65)		61 (66.3)	
Elective Cesarean section	22 (14.2)		19 (15.4)		14 (15.2)	
Nonelective Cesarean section	19 (12.3)		18 (14.6)		13 (14.1)	
Gestational diabetes		22		6		4
No	125 (80.6)		110 (89.4)		83 (90.2)	
Yes	8 (5.2)		7 (5.7)		5 (5.4)	

Maternal antibiotics by 4-months postpartum[2]		25		17		
No	113 (72.9)		92 (74.8)		92 (100)	
Yes	17 (11)		14 (11.4)		0 (0)	
Peripartum antibiotics		15		8		5
No	69 (44.5)		54 (43.9)		39 (42.4)	
Yes	71 (45.8)		61 (49.6)		48 (52.2)	
Infant feeding behavior		24		15		
Exclusively breastfed	109 (70.3)		87 (70.7)		75 (81.5)	
Combination fed	22 (14.2)		21 (17.1)		17 (18.5)	
Gestational age at delivery	39.2 [30 - 42.4]		39.2 [30 - 42.4]		39 [30 - 42.1]	
Infant sex		8		6		4
Female	73 (47.1)		57 (46.3)		41 (44.6)	
Male	74 (47.7)		60 (48.8)		47 (51.1)	
Postpartum collection week	6.6 [3.7 - 12]		6.6 [4.1 - 12]		6.6 [4.1 - 12]	

[a]The Kruskal Wallis rank-sum test or Fisher's exact test was used to test for differences in subject characteristics between groups

[b]Only postpartum antibiotic exposure differed significantly between groups

Table S2. Associations between maternal characters and BMT membership using multinomial logistic regression models

	OR (95% CI)[a]		
**Variable**	**BMT1 vs. BMT2**	**BMT3 vs. BMT2**	**BMT4 vs. BMT2**
Prepregnancy BMI	1.10 (1.01, 1.20)	1.11 (1.01, 1.22)	1.01 (0.91, 1.13)
Weight gain during pregnancy (per 10 lbs)	0.64 (0.43, 0.95)	1.08 (0.71, 1.65)	0.80 (0.53, 1.21)
Parity	0.99 (0.57, 1.73)	1.74 (1.02, 2.97)	1.49 (0.87, 2.54)
Delivery mode			
Vaginal (reference)			
Elective Cesarean section	1.74 (0.49, 6.14)	2.28 (0.63, 8.23)	0.72 (0.16, 3.28)
Nonelective Cesarean section	0.93 (0.29, 3.03)	0.61 (0.14, 2.59)	0.30 (0.06, 1.53)
Maternal prenatal antibiotics	1.07 (0.33, 3.44)	0.68 (0.16, 2.94)	1.35 (0.39, 4.64)
Maternal peripartum antibiotics	0.65 (0.27, 1.56)	1.17 (0.45, 3.08)	0.48 (0.19, 1.23)
Maternal antibiotics by 4-months postpartum	0.69 (0.18, 2.68)	0.73 (0.16, 3.22)	0.76 (0.19, 2.98)
Gestational age at delivery	0.87 (0.70, 1.07)	0.97 (0.76, 1.24)	1.06 (0.83, 1.37)
Postpartum collection week	1.10 (0.70, 1.72)	1.23 (0.76, 1.98)	1.50 (0.96, 2.33)

	OR (95% CI)[b]		
**Variable**	**BMT1 vs. BMT2**	**BMT3 vs. BMT2**	**BMT4 vs. BMT2**
Prepregnancy BMI	1.13 (1.02, 1.24)	1.12 (1.01, 1.25)	1.05 (0.94, 1.18)
Weight gain during pregnancy (per 10 lbs)	0.66 (0.44, 1.00)	1.20 (0.77, 1.88)	0.78 (0.49, 1.24)
Parity	0.84 (0.44, 1.58)	1.57 (0.81, 3.04)	1.37 (0.71, 2.64)
Postpartum collection week	1.25 (0.73, 2.11)	1.54 (0.87, 2.72)	1.72 (1.00, 2.95)

	OR (95% CI)[c]		
**Variable**	**BMT1 vs. BMT2**	**BMT3 vs. BMT2**	**BMT4 vs. BMT2**
Prepregnancy BMI	1.09 (0.98, 1.20)	1.08 (0.97, 1.20)	0.97 (0.85, 1.11)
Weight gain during pregnancy (per 10 lbs)	0.62 (0.38, 1.01)	1.17 (0.71, 1.94)	0.63 (0.36,1.09)
Parity	0.75 (0.37, 1.54)	0.98 (0.44, 2.19)	1.09 (0.50, 2.37)
Postpartum collection week	1.41 (0.75, 2.65)	1.60 (0.83, 3.09)	1.81 (0.96, 3.41)

[a]Univariate analyses

[b]Analyses adjusted for prepregnancy BMI, weight gain during pregnancy, parity, and postpartum collection week (n = 123)

[c]Analyses restricted to women known to have not received antibiotics before 4 months postpartum, and adjusted for prepregnancy BMI, weight gain during pregnancy, parity, and postpartum collection week (n = 92)

Table S3. Breast milk alpha diversity associations

Variable	Coefficient[a]	*p*-value[a]
Age	-0.0094	0.69
Prepregnancy BMI	-0.016	0.43
Weight gain during pregnancy (per 10 lbs)	0.21	0.039
Parity	-0.016	0.90
Delivery mode		
Vaginal (reference)		
Elective cesarean section	0.26	0.43
Nonelective cesarean section	0.15	0.66
Maternal prenatal antibiotics	0.16	0.61
Maternal peripartum antibiotics	0.33	0.16
Maternal antibiotics by 4-months postpartum	0.78	0.034
Infant feeding behavior		
Exclusively breastfed (reference)		
Combination fed	0.11	0.74
Gestational age at delivery	0.013	0.82
Infant sex		
Female (reference)		
Male	0.08	0.74
Postpartum collection week	-0.32	0.0033

[a]Univariate analyses

Table S4. Associations with breast milk microbial community structure

Variable	*p*-value[a]
Age	0.14
Prepregnancy BMI	0.38
Weight gain during pregnancy (lbs)	0.076
Parity	0.060
Delivery mode	0.27
Maternal prenatal antibiotics	0.84
Maternal peripartum antibiotics	0.58
Maternal antibiotics by 4-months postpartum	0.64
Infant feeding behavior	0.93
Gestational age at delivery	0.15
Infant sex	0.60
Postpartum collection week	0.057
**Variable**	***p*-value[b]**
Weight gain during pregnancy (lbs)	0.055
Parity	0.076
Postpartum collection week	0.073
**Variable**	***p*-value[c]**
Weight gain during pregnancy (lbs)	0.036
Parity	0.47
Postpartum collection week	0.095

[a]Univariate analyses

[b]Analyses adjusted for weight gain during pregnancy, parity, and postpartum collection week (N = 123)

[c]Analyses restricted to women known to have not received antibiotics before 4 months postpartum, and adjusted for weight gain during pregnancy, parity, and postpartum collection week (n = 92)
